# CD163+ Tumor-Associated Macrophages Correlated with Poor Prognosis and Cancer Stem Cells in Oral Squamous Cell Carcinoma

**DOI:** 10.1155/2014/838632

**Published:** 2014-05-06

**Authors:** Ke-Fei He, Lu Zhang, Cong-Fa Huang, Si-Rui Ma, Yu-Fan Wang, Wei-Ming Wang, Zhi-Li Zhao, Bing Liu, Yi-Fang Zhao, Wen-Feng Zhang, Zhi-Jun Sun

**Affiliations:** ^1^The State Key Laboratory Breeding Base of Basic Science of Stomatology and Key Laboratory of Oral Biomedicine Ministry of Education, Wuhan University, Wuhan 430079, China; ^2^Department of Oral Maxillofacial-Head Neck Oncology, School and Hospital of Stomatology, Wuhan University, Wuhan 430079, China

## Abstract

Tumor-associated macrophages (TAMs) play an important role in the progression and prognostication of numerous cancers. However, the role and clinical significance of TAM markers in oral squamous cell carcinoma (OSCC) has not been elucidated. The present study was designed to investigate the correlation between the expression of TAM markers and pathological features in OSCC by tissue microarray. Tissue microarrays containing 16 normal oral mucosa, 6 oral epithelial dysplasia, and 43 OSCC specimens were studied by immunohistochemistry. We observed that the protein expression of the TAM markers CD68 and CD163 as well as the cancer stem cell (CSC) markers ALDH1, CD44, and SOX2 increased successively from the normal oral mucosa to OSCC. The expressions of CD68 and CD163 were significantly associated with lymph node status, and SOX2 was significantly correlated with pathological grade and lymph node status, whereas ALDH1 was correlated with tumor stage. Furthermore, CD68 was significantly correlated with CD163, SOX2, and ALDH1 (*P* < 0.05). Kaplan-Meier analysis revealed that OSCC patients overexpressing CD163 had significantly worse overall survival (*P* < 0.05). TAM markers are associated with cancer stem cell marker and OSCC overall survival, suggesting their potential prognostic value in OSCC.

## 1. Introduction

Head and neck squamous cell carcinoma (HNSCC) is one of the most prevalent cancers and a major cause of mortality in patients with cancer worldwide. It accounts for 6% of all cancer cases and approximately 6,50,000 new cases every year [1]. Oral squamous cell carcinoma (OSCC) is the most frequent cancer of the head and neck area, accounting for 90% of HNSCC cases. It is characterized by high cervical lymph node metastasis and poor prognosis. The 5-year survival rate of patients with advanced OSCC has improved only marginally over the past three decades [1, 2].

Currently, cancer stem cells (CSCs, also called cancer-initiating cells) are gaining increasing attention in oncology research [3]. CSCs are a population of cancer cells with the abilities of self-renewal, differentiation, and tumor generation in immunodeficient mice [4]. CSCs are the major cause of tumor initiation, invasion, metastasis, and drug resistance and play an important role in tumors. Among the numerous identified CSC markers, aldehyde dehydrogenase 1 (ALDH1) is a cytosolic isoform of ALDH, and high expression of ALDH1 is a predictor of poor clinical outcome in many cancers [5]. CD44, an integral cell membrane glycoprotein, plays an important role in adhesion and migration [6]. In addition, the SRY-related HMG-box gene 2 (SOX2) is a promising new marker for CSCs, which plays multiple roles in stem cell maintenance and tumorigenesis [7, 8]. Overexpressed SOX2 has been associated with the progression in the squamous cell carcinomas [8, 9]. Thus, we aimed to investigate the expression of SOX2, ALDH1, and CD44 in OSCC.

Numerous pieces of evidence have suggested that the tumor microenvironment plays a critical role in tumor development, particularly CSC [10]. Nowadays, as one of the major components of the microenvironment, tumor-associated macrophages (TAMs, also called tumor-infiltrating macrophages) have been shown to promote tumor progression by influencing tumor invasion, migration, and angiogenesis [11–13]. Macrophages constitute a major component of the infiltrates in most solid tumors [14]. Macrophages exist in two distinct polarized states: one is the classically activated (M1) state and the other is the alternatively activated (M2) state. M1 macrophages possess antitumor activity, whereas M2 macrophages promote tumor invasion and metastasis [15, 16]. However, most TAMs have a M2-like phenotype. CD68 is a pan-macrophage marker frequently used as a marker for TAMs, and CD68 recognizes both tumoricidal M1 and anti-inflammatory M2 macrophages. CD163, a marker of M2 macrophages, has been studied in several aggressive tumors, and the increased expression of CD163 was significantly associated with a poor overall survival (OS) in various cancers [15–18]. To the best of our knowledge, CD163 has not been evaluated as an M2 macrophage marker in primary OSCC. Therefore, in the present study, we examined the expression of CD68 and CD163 in OSCC tissues. Over the past few years, an increasing number of studies have highlighted the interaction between CSCs and their niche microenvironment. Moreover, to the best of our knowledge, macrophages are the most important ancillary cells regulating CSC activities. However, their correlation with OSCC remains unclear.

In this study, we examined the expression of TAM and CSC markers in OSCC using tissue microarray and analyzed the association among these makers. In addition, we evaluated the association of the expression of TAM and CSC markers with pathological features and clinical outcomes to clarify their roles in OSCC prognosis.

## 2. Materials and Methods

### 2.1. Patient Samples and Custom Made Tissue Array

The OSCC tissue microarrays of humans used in this study were obtained from 2008 to 2009 in the Department of Oral and Maxillofacial Surgery, School and Hospital of Stomatology, Wuhan University. With the approval of the Wuhan University Medical Ethics Committee (principle investigator: Zhi-Jun Sun), informed written consent was obtained from each patient. The procedures were performed in accordance with the National Institutes of Health guidelines regarding the use of human tissues. Hospital records, pathology reports, and histology slides of all patients were retrieved and reviewed. Whereas the clinical stages of the TSCC were classified according to the guidelines of the International Union against Cancer (UICC 2002), the grading scheme of the World Health Organization was used to determine the histologic grading. The medium follow-up period was 24 months (range from 12 to 43 months). Custom made tissue microarrays (T12–412) using oral cancer specimens contained above formalin-fixed, paraffin embedded tissues. The oral cancer cohort consisted of 17 normal oral mucosa, 7 oral epithelial dysplasia, and 43 oral cancers specimens from 43 patients. Clinical information including T category, lymph node metastasis, TNM stage, and histologic grade has been previously described [19].

### 2.2. Immunohistochemistry (IHC)

Immunohistochemical studies of the human OSCC tissue microarrays were done using the following antibodies: CD68 (ZM-0464, dilution 1 : 50; Zymed); CD163 (CWP107-1, dilution 1 : 50; CWBiotech); CD31 (number 2540, dilution 1 : 750; Epitomics); SOX2 (AM-2048a, dilution 1 : 100; Abgent); CD44 (15675-1-AP, dilution 1 : 100; Proteintech); ALDH1 (15910-1-AP, dilution 1 : 100; Proteintech), which was stained in serial-cut tissue array sections as previously described [20].

All sections were air-dried overnight at 60°C; then antigen retrieval was done using a 0.01 M citric acid buffer solution (pH 6.0). After cooling down to room temperature, sections were incubated in 3% hydrogen peroxide to quench the endogenous peroxidase activity and treated with 10% normal goat serum. Sections were incubated at 4°C overnight within primary antibody, followed by second antibody at room temperature. Thereafter, sections were incubated within an avidin-biotin-peroxidase reagent for another 20 min. After sections adequate elusion with PBS, Diaminobenzidine as well as a counterstaining with haematoxylin resulted in the visualization of the immunostaining.

### 2.3. Scoring System

Slices were scanned using an Aperio ScanScope CS scanner (Vista, CA, USA) with background substrate for each slice and quantified using Aperio Quantification software (Version 9.1) for membrane, nuclear, or pixel quantification as previously described [20]. An area of interest was selected either in the epithelial or the cancerous area for scanning and quantification. Histoscore of membrane and nuclear staining was calculated as a percentage of different positive cells using the formula (3+) × 3 + (2+) × 2 + (1+) × 1. Histoscore of pixel quantification was calculated as total intensity/total cell number. The threshold for scanning of different positive cells was set according to the standard controls provided by Aperio.

### 2.4. Statistical Analysis of Clinical Features

Statistical data analysis was performed with GraphPad Prism 5.03 for Windows (GraphPad Software, Inc., La Jolla, CA, USA) statistical packages. One-way ANOVA followed by the post-Tukey's or Bonferroni's multiple comparison tests was used to analyze the differences in immunostaining and protein levels among each group. Two-tailed Pearson's statistics was used for correlated expression of CD68 and CD163, with SOX2, CD44, and ALDH1 after confirmation of the sample with Gaussian distribution. Survival curves were plotted using the method of Kaplan-Meier and the significance of observed differences was assessed with log-rank test. The data were present as mean ± SEM with a difference of *P* < 0.05 considered statistically significant.

### 2.5. Hierarchical Clustering and Data Visualization

For further cluster analysis, the staining scores obtained from immunohistochemistry were recorded in Microsoft Excel format and converted into scaled values centered on zero. The hierarchical analysis was done using Cluster program with average linkage based on Pearson's correlation coefficient. And the results were visualized using the TreeView program. The clustered data were arranged with markers on the horizontal axis and tissue samples on the vertical axis. Two biomarkers with a close relationship are located next to each other.

## 3. Results

### 3.1. Expression of TAM Markers in Normal Oral Mucosa, Oral Epithelial Dysplasia, and OSCC Specimens

**Figure 1:**
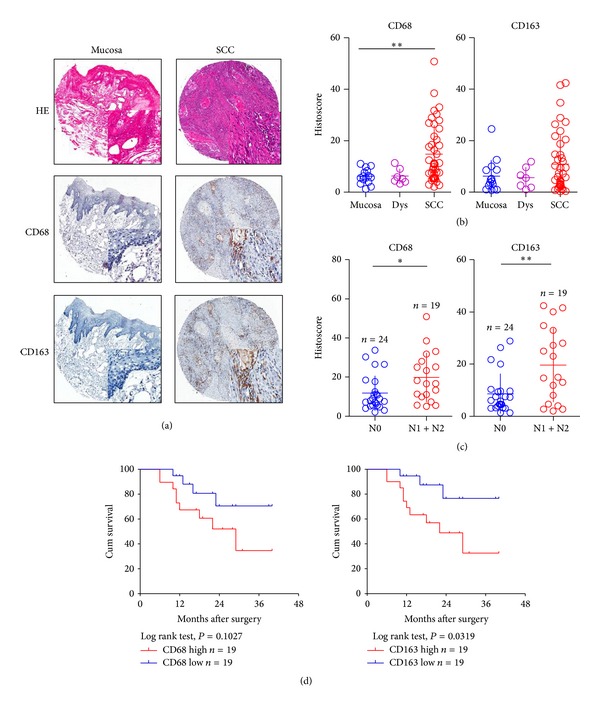
Human OSCC tissue array analysis revealed that CD68 and CD163 were overexpressed in human OSCCs. (a) Representative HE staining and immunohistochemical staining (IHC) of CD68 and CD163 in human oral cancer tissue (right) compared with normal oral mucosa (left) (scale bars = 100 *μ*mol). (b) Quantitative histoscore of CD68 and CD163 expression in normal oral mucosa, oral epithelial dysplasia, and human oral cancer; CD68 levels were significantly higher when compared with normal oral mucosa (mean ± SEM; *, *P* < 0.05; **, *P* < 0.01). (c) The expressions of CD68 and CD163 were correlated with lymph node status of human oral cancer (quantification was done using Aperio nuclear quantification software, and statistics was calculated using Graph Pad Prism 5. Mean ± SEM; *P < 0.05; **P < 0.01). (d) Overall survival of the OSCC patients with CD68 and CD163 expression calculated and presented by Kaplan-Meier analysis, and CD163 expression was significantly correlated with overall survival (*P* = 0.0319, *n* = 38).

To investigate whether the expression of TAM markers varies in OSCC, we assessed the expression of two TAM markers, CD68 and CD163, in OSCC using tissue microarray. Our analysis revealed that CD68 and CD163 are expressed in the cytoplasm and frequently observed in OSCC samples (Figure 1). The mean expression score of CD68 in normal oral mucosa was 4.99 ± 0.38, and its expression was significantly different as compared with that in oral epithelial dysplasia (5.62 ± 1.86) and OSCC (17.59 ± 1.91). Additionally, a significant difference between CD68 staining in OSCC and normal oral mucosa was observed (*P* < 0.01). Furthermore, the expression of CD163 in OSCC was mainly observed in the cytoplasm and the mean expression scores were 18.33 ± 1.29, 5.14 ± 0.52, and 4.80 ± 0.53 in OSCC, oral epithelial dysplasia, and normal oral mucosa, respectively; however, the difference between CD163 staining in the tumor and normal oral mucosa was not significant (*P* > 0.05, Figure 1(b)).

### 3.2. Correlation among the Expression of TAM Markers, Pathological Features, and Stage of Disease

On further evaluation of the relationships among TAM markers and clinical pathological features, we found that CD68 was significantly associated with lymph node status (N0 and N1 + N2; *P* < 0.05; Figure 1(c)) and CD163 (N0 and N1 + N2; *P* < 0.01; Figure 1(c)). However, we did not find that the expression of CD68 and CD163 was correlated with tumor stage (T1 to T3) or pathological grade (G I to G III; *P* > 0.05; Supplementary Figure 1 (see Supplementary Material available online at http://dx.doi.org/10.1155/2014/838632) in our research, and a large sample of OSCC tissues with follow-up will be collected to further confirm the correlation between CD68 and CD163 with tumor stage and pathological grade.

### 3.3. Expression of CSC Markers in Normal Oral Mucosa, Oral Epithelial Dysplasia, and OSCC

Although we have previously studied the expression of SOX2, ALDH1, and CD44 in OSCC, their expression and correlation with macrophage-associated markers using automated analysis on a serial section of tissue microarrays has not been formerly evaluated. Following analysis, we observed that the mean expression scores of SOX2 were 117.6 ± 2.8 (*n* = 43), 205.9 ± 10.7, and 40.2 ± 5.9 in OSCC, oral epithelial dysplasia, and normal mucosa, respectively. In addition, a significant difference in SOX2 staining was demonstrated between OSCC, oral epithelial dysplasia, and normal oral mucosa (*P* < 0.05). The mean expression scores of ALDH1 were 78.4 ± 1.9, 69.1 ± 6.4, and 41.6 ± 3.1 in OSCC, oral epithelial dysplasia, and normal mucosa, respectively; furthermore, a significant difference between ALDH1 staining in OSCC and normal oral mucosa was observed (*P* < 0.01). The mean expression scores of CD44 were 268.4 ± 13.5, 240.8 ± 20.4, and 191.6 ± 18.3 in OSCC, oral epithelial dysplasia, and normal mucosa, respectively; CD44 staining too demonstrated a significant difference between the OSCC and normal oral mucosa (*P* < 0.001).

### 3.4. Correlation between the Expression of CSC Markers, Pathological Features, and Stage of Disease

**Figure 2:**
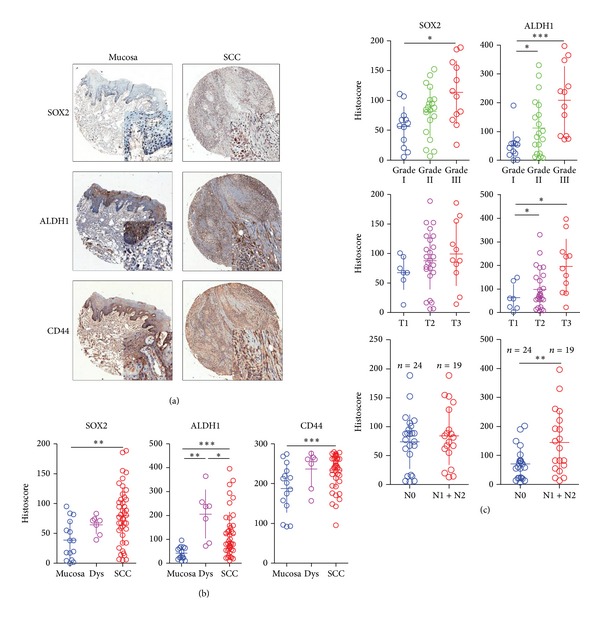
Human OSCC tissue array analysis revealed that SOX2, ALDH1, and CD44 were overexpressed in human OSCCs. (a) Representative HE staining and immunohistochemical staining (IHC) of SOX2, ALDH1, and CD44 in human oral cancer tissue (right) compared with normal oral mucosa (left) (scale bars = 100 *μ*mol). (b) Quantitative histoscore of SOX2, ALDH1, and CD44 expression in normal oral mucosa, oral epithelial dysplasia, and human oral cancer, and SOX2, ALDH1, and CD44 levels were significantly higher when compared with normal oral mucosa (mean ± SEM; *, *P* < 0.05; **, *P* < 0.01; ***, *P* < 0.001; One-way ANOVA). (c) SOX2 and ALDH1 were significantly associated with pathological grade, and ALDH1 was significantly correlated with tumor stage and lymph node status (quantification was done using Aperio nuclear quantification software, and statistics was calculated using Graph Pad Prism 5. Mean ± SEM; *P < 0.05; **P < 0.01; ***P < 0.001).

Figure 2 and Supplementary Figure 2 show the association of several clinicopathological factors with SOX2, ALDH1, and CD44. The expression of SOX2 was significantly correlated with pathological grade (G I and G III, *P* < 0.05) but not with tumor stage (T1–T3) and lymph node status (N0 and N1 + N2, *P* > 0.05); ALDH1 was significantly correlated with tumor stage (T1–T3) and pathological grade (G I–G III, *P* < 0.05) but not with lymph node status (N0 and N1 + N2, *P* > 0.05); and CD44 was not significantly correlated with either the tumor stage (T1 to T3), pathological grade (G I–III), or lymph node status (*P* > 0.05).

### 3.5. Survival Analysis

In addition, follow-up information was available for 43 patients with OSCC, ranging from 11 months to 40 months (21.6 ± 1.2). At the end of this study, 5 patients were lost during follow-up period, 25 patients were alive, and 15 patients had recurrence with 13 of them dead of cancer. The 3-year overall survival is 51.2 and and disease-free survival rate was 47.1%. Figure 1(d) shows the survival curves stratified on the basis of the expression of CD68 and CD163. The overall survival was calculated by median histoscore of CD68 and CD163 in OSCC, respectively. The expression of CD68 and CD163 can distinctly indicate the survival of patients with OSCC; the Kaplan-Meier method indicated that, in patients, the cumulative rate of the expression of CD68 was not significantly correlated with overall survival (OS) (*P* = 0.1027, *n* = 38), whereas that of CD163 was significantly correlated with OS (*P* = 0.0319, *n* = 38).

### 3.6. The Relationship between the Expressions of CD68, CD163, SOX2, ALDH1, and CD44

**Figure 3:**
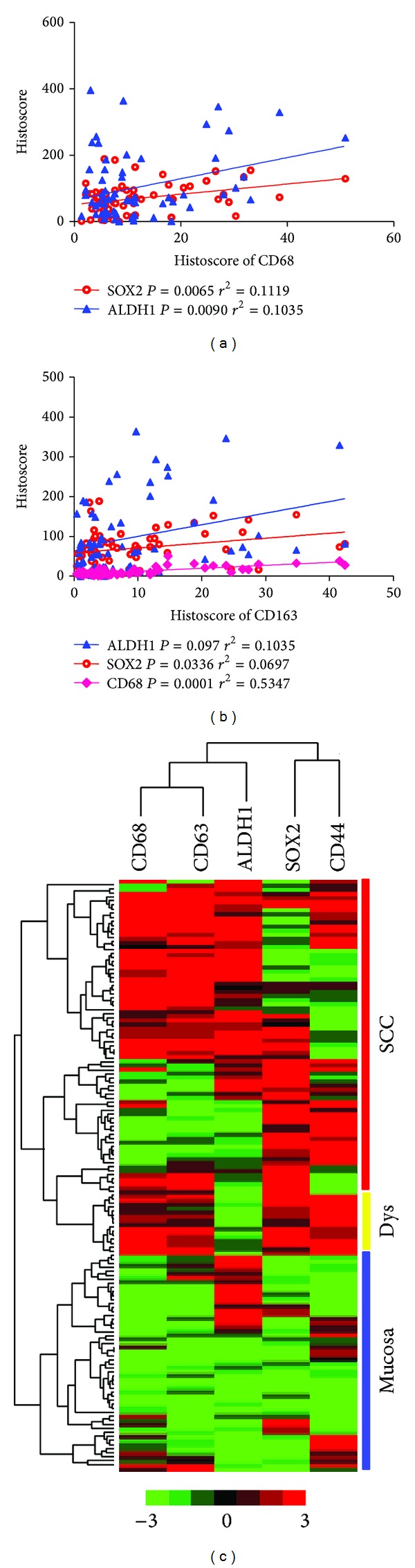
Correlation of CD68 and CD163 with SOX2, ALDH1, and CD44 in human OSCC tissue array. (a) The expression of CD68 had significant correlations with SOX2 (*P* = 0.0065, *r* ^2^ = 0.1119) and ALDH1 (*P* = 0.0090, *r* ^2^ = 0.1035) by using the Pearson's correlation coefficient test in human OSCC tissue array. (b) The expression of CD163 was closely correlated with SOX2 (*P* = 0.0336, *r* ^2^ = 0.0697), ALDH1 (*P* = 0.0097, *r* ^2^ = 0.1035), and CD68 (*P* = 0.0001, *r* ^2^ = 0.5347) in human OSCC tissue array. (c) Hierarchical clustering of CD68 and CD163 with SOX2, ALDH1, and CD44 in human OSCC tissue array. It shows that the expression of tumor-associated macrophage markers CD68 and CD163 is more close to expression of SOX2.

To assess the correlation between the expression of TAM and CSC markers in human OSCCs, we stained the tumor sections from human tissue arrays for OSCC with antibodies for CD68, CD163, SOX2, ALDH1, and CD44 and compared them with normal oral mucosa samples. Using the Pearson correlation coefficient test, we observed that increased protein levels of CD68 had significant correlations with SOX2 (*P* = 0.0065, *r* ^2^ = 0.1119) and ALDH1 (*P* = 0.0090, *r* ^2^ = 0.1035) (Figure 3(a), Table 1). In addition, CD163 was closely correlated with SOX2 (*P* = 0.0336, *r* ^2^ = 0.0697), ALDH1 (*P* = 0.0097, *r* ^2^ = 0.1035), and CD68 (*P* = 0.0001, *r* ^2^ = 0.5347, Figure 3(b), Table 1). Hierarchical clustering demonstrated that the expression of TAM markers, CD68 and CD163, is more closely associated with the expression of SOX2 (Figure 3(c)).

## 4. Discussion

In recent years, CSCs are often relatively quiescent and present resistance characteristics to conventional drugs. Increasing number of studies are being conducted today with a focus on CSCs and their niche microenvironment because signaling from the niche microenvironment regulates CSC function [21]. Recently, TAMs have been reported to play a key role in maintaining the undifferentiated state of CSCs and promote tumor progression [22]. Despite the recent findings, the association and roles between TAMs and CSCs and the activation of carcinogenesis in OSCC remain largely unknown.

In this study, we have studied the expression of SOX2, ALDH1, and CD44 in OSCC using tissue microarray and found that all of them were overexpressed in OSCC. To date, little is known about its association with TAMs in oral cancer carcinogenesis. TAMs are known to play a significant role in tumor progression. Although different macrophage subtypes perform specific functions, their roles and markers have not been entirely investigated in malignant tumors [23]. Previous studies have identified tumor promoting roles in TAMs resembling M2 macrophages [24]. CD68 is a pan-macrophage marker frequently used as a marker for TAMs. CD163 is regarded as a highly specific macrophage marker for M2 macrophages, and increased CD163 was significantly associated with a poor overall survival in cancers [15–18]. In the present study, we demonstrate an increased expression of CD68 and CD163 in the cytoplasm of OSCC compared with that in the normal oral mucosa. Our study was in accordance with the researches in bladder and breast cancer, suggesting that CD68 and CD163 are important diagnostic and prognostic factors in OSCC.

Furthermore, previous studies have clarified the macrophages secrete growth and other factors that permeate the cancer stem cell niche to promote survival and self-renewal of stem cells. However, the precise role of TAMs mediate tumorigenesis through regulation of CSCs remains unclear. Our results demonstrate a significant correlation of CD68 with SOX2 and ALDH1 using the Pearson correlation coefficient test and of CD163 with SOX2, ALDH1, and CD68. Hierarchical clustering demonstrates a close association between the expression of TAM markers CD68 and CD163 and the CSC marker ALDH1. Takakura identified that the macrophage-derived factors such as milk fat globulin epidermal growth factor-8 and IL-6 play a critical role in the tumorigenic activities of colon CSCs (CD44+ALDH1+) [21]. In addition, the synthase HAS2 plays a critical role in CD44+/CD24/ESA+ breast CSCs by enhancing the interaction between CSCs and TAMs by inducing the secretion of PDGF-BB in TAMs [25]. Recently, the CD44 signaling pathway, which promotes tumorigenicity in colorectal cancer, was identified between TAM and CD44-positive cancer cells [26]. In addition, the study by Sanford et al. revealed that preventing the recruitment of macrophages results in a significant reduction in tumor infiltration and CD44+ALDH+ pancreatic CSCs [27]. Therefore, further investigation is needed to understand the specific mechanisms between TAMs and the initiation of CSCs in OSCC.

This study determined that positive expressions of CD68 and CD163 were significantly associated with the aggressive behavior of OSCC, including lymph node status. In breast cancer, the tumor stroma was infiltrated by CD163+ and CD68+ macrophages, CD163+ macrophages positively correlated with higher grade and larger tumor size, and CD68+ macrophages positively correlated with tumor size [18]. The proportions of CD163+ macrophages among CD68+ reflect the proportion of macrophages polarized to M2 phenotype, which was correlated with the histological grade in gliomas [28], and these findings were in accordance with our present study. Following these findings, our results indicate that CD68 and CD163 may play important roles in carcinogenesis and progression in the patients of oral cancer.

A feedback system exists between CSCs and TAMs, CSCs render the progress of the tumor by recruiting TAMs through blood vasculature, and TAMs in turn release chemokines to maintain CSC quiescence. Cancer cells expressing CD163 were associated with poor prognosis in patients with breast cancer [29] and rectal cancer [30]. The density of peritumoral CD68+ cells was associated with poor recurrence-free survival and overall survival [31]. More importantly, CD68 in the tumor stroma was an independent prognostic factor for reduced breast cancer specific survival [28]. The results of these studies were in accordance with our present study that demonstrates that the expression of CD163 significantly correlated with poor overall survival. Since the sample size is limited in this study, a large scale of OSCC tissue with follow-up will be collected to further confirm the diagnostic and prognostic role of TAMs in OSCC progression. Interestingly, recent studies [32, 33] showed that TAMs can increase the pluripotency of the SOX2 gene through EGFR by activating STAT3 signaling; inhibition of EGFR or STAT3 will improve chemosensitivity by preventing SOX2 upregulation, indicating that TAMs inhibition may be a novel treatment strategy for CSC therapy.

In conclusion, our findings reveal that the TAM markers CD68 and CD163 were significantly associated with the aggressive behavior of OSCC, including N classification, and significantly correlated with the CSC markers SOX2, ALDH1, CD44, and CD163, whose expression was significantly correlated with OS. However, further studies are required to identify the specific crosstalk between OSCC CSCs and TAMs.

## Supplementary Material

Supplementary Figure 1: The correlation between the expression of CD68 and CD163 with pathological grade and tumor stage in OSCC.
Supplementary Figure 2: The correlation between the expression of CD44 with pathological grade, tumor stage and lymph node status in OSCC; Overall survival of the OSCC patients with SOX2, ALDH1 and CD44 expression calculated and presented by Kaplan–Meier analysis.

## Figures and Tables

**Table 1:** Pearson's correlation coefficient test analyses of the array immunostainings of CD68, CD163, ALDH1, SOX2, and CD44 in OSCC.

Markers	CD163	SOX2	ALDH1	CD44
CD68	*P* = 0.0090, *R* ^2^ = 0.1035	*P* = 0.0065, *R* ^2^ = 0.1119	*P* = 0.0090, *R* ^2^ = 0.1035	*P* = 0.1067
CD163		*P* = 0.0065, *R* ^2^ = 0.1119	*P* = 0.0090, *R* ^2^ = 0.1035	*P* = 0.1323
SOX2			*P* = 0.2859	*P* = 0.1463
ALDH1				*P* = 0.6618
