# Supplementary figures and images for: CD163+ Tumor-Associated Macrophages Correlated with Poor Prognosis and Cancer Stem Cells in Oral Squamous Cell Carcinoma
Source: Biomed Res Int. 2014 May 6;2014:838632. doi: 10.1155/2014/838632 (PMC4032721; doi:10.1155/2014/838632)

Supplementary Figure 1

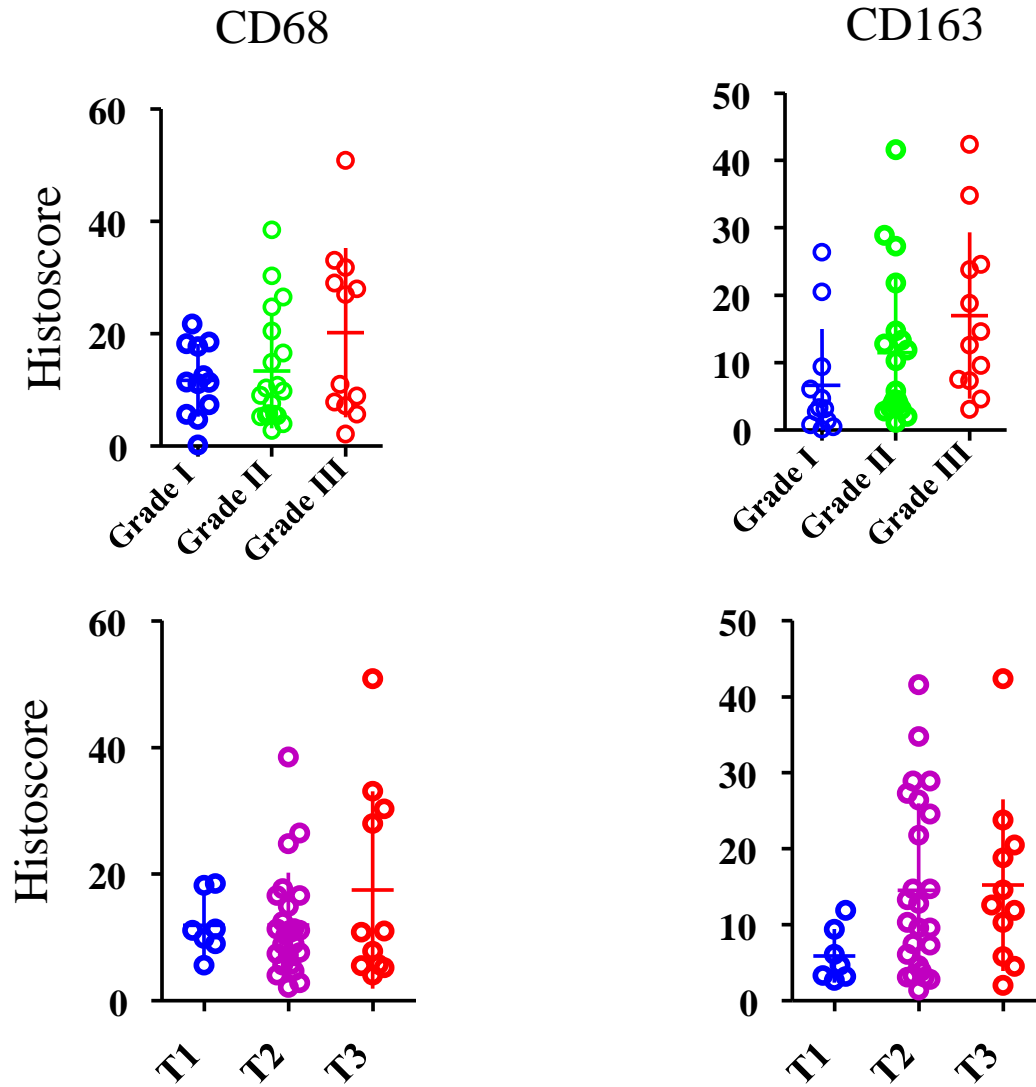

# Supplementary Figure 2

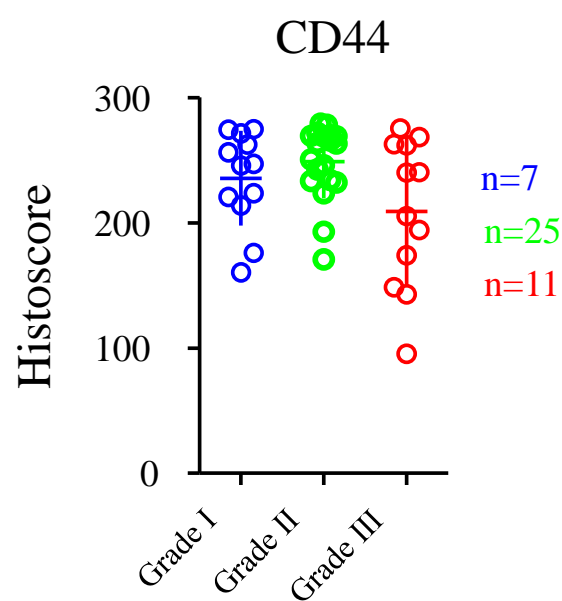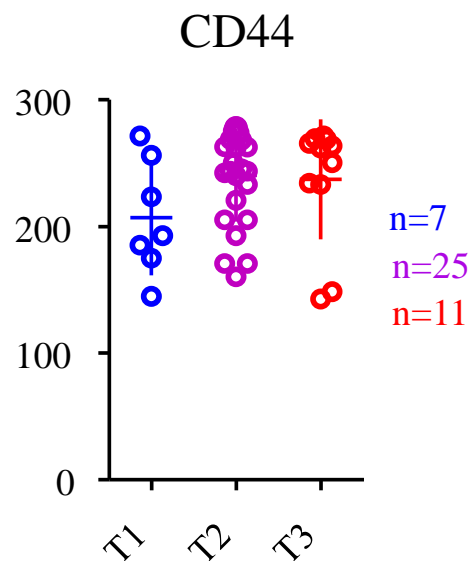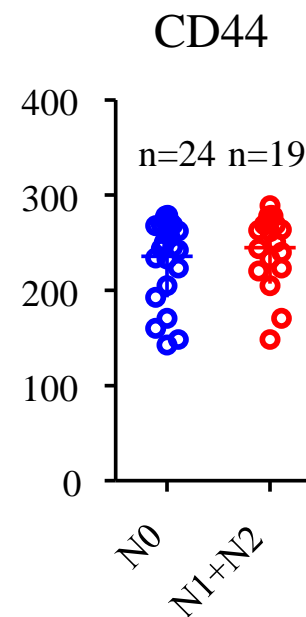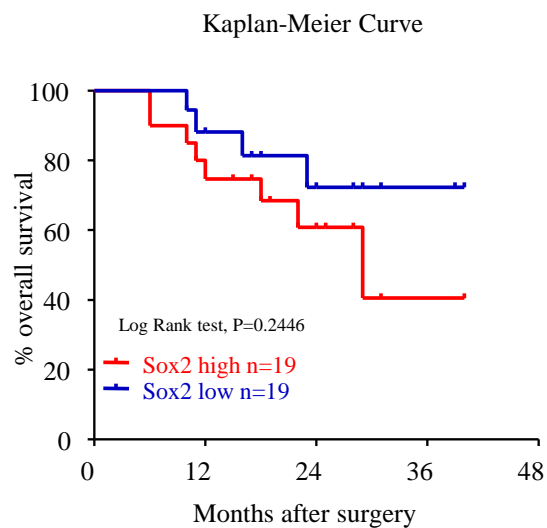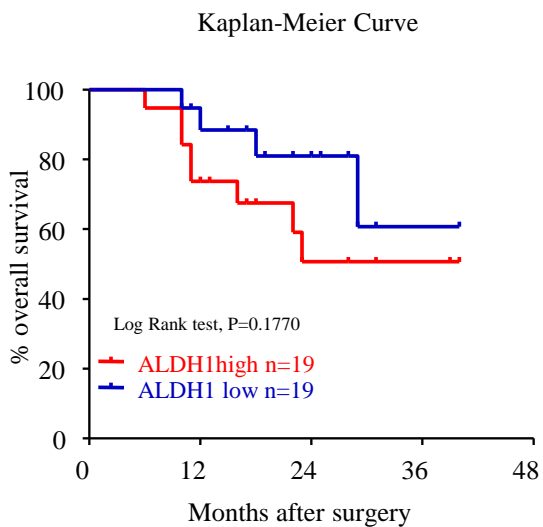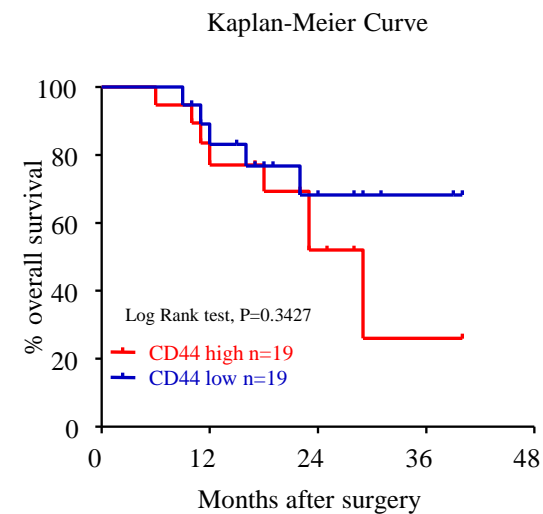

Supplement: Supplementary file 1 — Supplementary Figure 1: The correlation between the expression of CD68 and CD163 with pathological grade and tumor stage in OSCC. Supplementary Figure 2: The correlation between the expression of CD44 with pathological grade, tumor stage and lymph node status in OSCC; Overall survival of the OSCC patients with SOX2, ALDH1 and CD44 expression calculated and presented by Kaplan–Meier analysis. [file 838632.f1.pdf]
